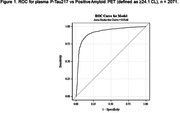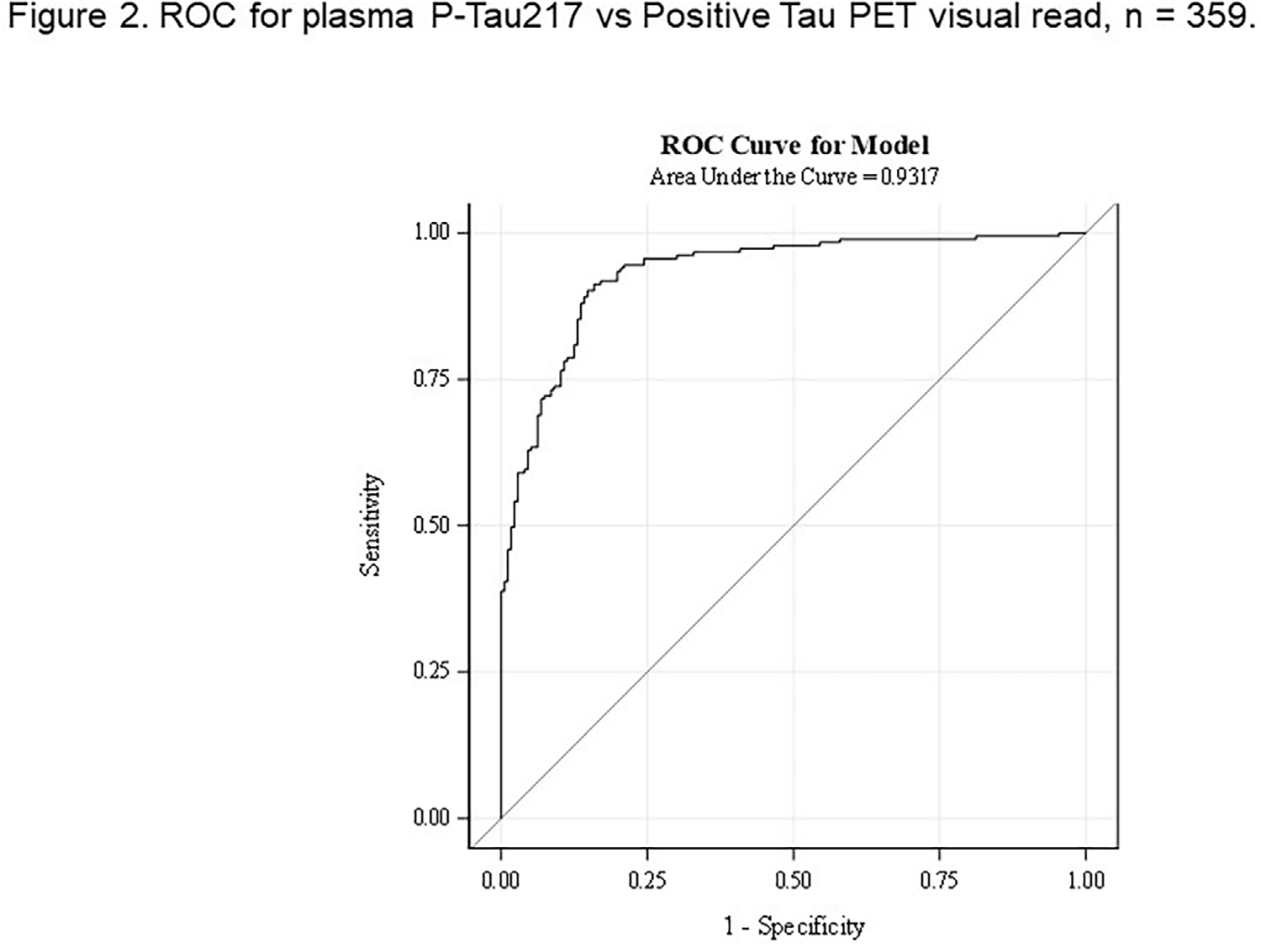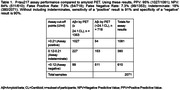# Clinical validation of the Eli Lilly SPX P‐tau217 blood‐based immunoassay as a laboratory‐developed test (LDT)

**DOI:** 10.1002/alz.095087

**Published:** 2025-01-09

**Authors:** Michael Hodsdon, Adam Abel, Antonio Chambers, Ming Lu, Amanda Morris, Michael Pontecorvo, Heinz Reiske, Emily C. Collins, Mark Mintun, Andrew E. Schade, Rose C. Beck

**Affiliations:** ^1^ Eli Lilly and Company, Indianapolis, IN USA

## Abstract

**Background:**

Blood‐based biomarkers have significant potential to aid in the diagnosis of Alzheimer’s disease (AD), providing a more accessible option than cerebrospinal fluid testing or positron emission tomography (PET). This study evaluated performance of a plasma P‐tau217 immunoassay for identifying AD pathology as detected by amyloid or tau PET.

**Method:**

Plasma samples from screened participants from the donanemab Phase 3 trial (NCT04437511) were analyzed using a chemiluminescent immunoassay for plasma P‐tau217 on the Quanterix SP‐X platform. A Receiver Operating Characteristic (ROC) curve explored the relationship between plasma P‐tau217 values and positive amyloid PET (Aβ+), defined as 24.1 Centiloid (CL). A two‐threshold approach was used to propose P‐tau217 cut‐points that result in sensitivity and specificity each ≥90%; <20% of cases falling between the two cut‐off values (classified as indeterminate). Similar evaluation was performed for P‐tau217 against tau PET in a subset of participants.

**Result:**

Plasma samples from 2071 participants (mean age 73.1 [SD = 6.4 years], Mini Mental State Examination mean 24.8 [SD = 2.5], 53.3% female, 50.7% *APOE* ε4 carriers, 65% Aβ+) were examined. The area under the curve (AUC) for the ROC of plasma P‐tau217 identifying Aβ+ participants was 0.915 (Figure 1). Stratification of P‐tau217 concentrations into three levels using two P‐tau217 cut‐points resulted in an upper‐level group with 95% positive predictive value (PPV) for Aβ+ and a low‐ level group with 84% negative predictive value (NPV) for Aβ‐, with a false positive rate of 7.5% and a false negative rate of 7.3% (Table 1). The remaining indeterminate group represented 18% of participant samples, of which 60% were Aβ+. Within this cohort, 359 participants had available tau PET results. Preliminary analysis revealed 93% ROC AUC for P‐tau217 against tau PET visual read (Figure 2).

**Conclusion:**

The presented plasma P‐tau217 assay has high concordance with amyloid PET for identification of amyloid pathology, indicating its potential utility in the diagnostic workup of AD. Preliminary analysis demonstrated a relationship between plasma P‐tau217 and tau PET; however, additional work is needed to establish the assay’s diagnostic performance for tau pathology.